# Willingness to participate and Pay for a proposed national health insurance in St. Vincent and the grenadines: a cross-sectional contingent valuation approach

**DOI:** 10.1186/s12913-015-0806-3

**Published:** 2015-04-09

**Authors:** Rosmond Adams, Yiing-Jenq Chou, Christy Pu

**Affiliations:** Ministry of Health, Wellness and Environment, Ministerial Building, Halifax Street, Kingstown, St. Vincent and the Grenadines; Department of Public Health, National Yang Ming University, 155, Sec.2, Linong Street, Taipei, 112 Taiwan

**Keywords:** National health insurance, Willingness to pay, Contingent valuation, St. Vincent and the Grenadines

## Abstract

**Background:**

Numerous Caribbean countries are considering implementing National Health Insurance (NHI) and pooling resources to finance their health sectors. Based on this increased interest in health insurance, we investigated the willingness to participate and to pay for NHI in St. Vincent and the Grenadines, an upper-middle-income Caribbean country.

**Methods:**

Four hundred heads of household in St. Vincent and the Grenadines were interviewed in August 2012 and September 2012. The samples were selected through simple random sampling, including the stratification of rural, semiurban, and urban communities to ensure the representativeness of the sample. A contingent valuation method with a pretested interviewer-led questionnaire was used. Respondents were presented with a hypothetical NHI plan. Chi-squared analysis was performed to identify factors that are associated with the willingness to participate. Multiple logistic regression was used to explore the factors that influence respondents’ willingness to pay.

**Results:**

In total, 69.5% (n = 278) of the respondents indicated that they were willing to participate in the proposed NHI plan, of whom 72.3% were willing to pay for the first bid (EC$50). When the bid was reduced to EC$25, all of the remaining respondents who indicated they were willing to participate were willing to pay this lowered bid. Overall, the respondents were willing to pay EC$77.83 (US$28.83) per month for each person to enroll in the NHI plan. Age, income, and having some form of health insurance were significantly associated with a willingness to participate in the plan.

**Conclusions:**

A higher socioeconomic status was the principal determinant factor for the willingness to participate. This is similar to studies on developing economies. The government can use these findings to guide the successful implementation of the proposed NHI program. People with a lower socioeconomic status must be engaged from the start of and throughout the development process to enhance their understanding of and participation in the plan.

## Background

St. Vincent and the Grenadines is an archipelagic State in the Eastern Caribbean. The country is comprised of a main island, St. Vincent, and a chain of 32 islands and cays, the Grenadines. The total area of the country is 150 square miles (389 square kilometers). The country is an upper-middle-income microstate with all the inherent challenges, such as a narrow economic base and high vulnerability to external shocks and natural disasters. The mainstay of the economy is agriculture, but increasingly, tourism and other services, and construction have become important contributors to the economy. Based on the 2001 Population and Housing Census Report, the population of St. Vincent and the Grenadines stood at 106,253 [1]. There is informal labor participation in the agricultural, construction and retail sectors. In 2010 around 80% of Vincentian businesses were informal, micro or small enterprises, and 60% of all employed persons were working in micro and small enterprises [2]. According to the most recent data available from the government, in 2010, St. Vincent and the Grenadines’ labor force was approximately 63000 or 52% of the population [3].

In the late 1990s, numerous Eastern Caribbean countries, such as St. Vincent and the Grenadines, began conducting feasibility studies on the establishment of a National Health Insurance (NHI) program [4]. The initiative for a policy formulation stage of NHI in the Eastern Caribbean originated from the interest of various governments to find alternative ways to finance their health sectors for adopting universal health care coverage [5]. However, the momentum of implementing NHI seems to have been lost after the study phase was complete.

Policy dialogue and research began in St. Vincent and the Grenadines in 1995, with the country considering the implementation of NHI based on studies completed by the Health Economics Unit of the University of the West Indies [6]. In St. Vincent and the Grenadines, health care delivery and financing are based on an integrated model of public health. The Ministry of Health, Wellness and the Environment (MOHWE) plays the principal role in funding, managing, and providing health services throughout this multi island country [7]. As a provider of services, the government owns and operates most of the health care facilities.

The government budget principally finances the health sector, and accounts for more than two-thirds of the total financing that the health sector receives [8]. External sources, such as the Caribbean Development Bank (CDB) and the Pan American Health Organization, provide additional funding for health care, as do governments such as those of Taiwan and Cuba [9]. Private sector contributions and out-of-pocket spending also contribute to the financing of the health care system. The exact contribution from private sectors and from out-of-pocket spending are unknown, because data from the private sector are scant, and no population-wide household expenditure survey has ever been conducted. The World Health Organization estimates that nearly all of the private health expenditures in St. Vincent and the Grenadines are out of pocket, and that little or no private expenditure is pooled through insurance premiums [10].

The government of St. Vincent and the Grenadines does not provide health insurance coverage as a benefit, not even for public-sector employees. People with health insurance have enrolled privately, and some have formal employment-sponsored insurance, although this is rare and not required by law. One study conducted in 2009 for the CDB estimated that only 9.4% of the population had health insurance [11].

Health care accounted for only 7.7% of the total government expenditure in 2010 [12]. Recent data indicate a decreasing trend in the government’s contribution to the health sector. An extremely low percentage of GDP spent on health in St. Vincent and the Grenadines suggests that not enough resources are mobilized for health, that access to health care is insufficient, and that the quality of services may not be adequate. Because of the current global economic downturn many developing countries had to cut back spending in almost all sectors of their economy, including health. The government budgets of St. Vincent and the Grenadines were no exception [13]. Therefore, new and intuitive means of financing, sustaining, and improving health care must be developed because the method of financing health care in the past may no longer be the most suitable for finance the health sector, considering recent budgetary limitations and increased burden on health care due to the country’s changing epidemiological profile as the burden of chronic diseases increases [14].

We investigated the willingness to participate, and for those who were willing to participate, their willingness to pay for NHI in St. Vincent and the Grenadines. Numerous studies have focused on the willingness to pay for health insurance in developed countries [15-17]. Some insurance studies have focused on community-based health insurance (CBHI) in African countries [18-22]. However, few insurance studies have explored developing countries, particularly countries in the regions of the Caribbean.

In this study, we examined the socioeconomic, demographic, and health characteristics of the respondents. We selected the independent variables based on literature review [23-27].

The results of this study can provide evidence that will enable policy makers to enhance their understanding of the factors that determine people’s willingness to participate and to pay for NHI. This will allow the targeting of those who wish to participate, and will encourage participation among the less willing. The results can also serve as a reference for setting premium values, and for future policy decision making in other Caribbean countries, particularly those in the Eastern Caribbean, where the same model of health care exists, and the economies are relatively similar.

## Methods

### Sampling and sample size

The current study was conducted in St. Vincent and the Grenadines during August 2012 and September 2012. The survey was administered in 15 locations in St. Vincent and the Grenadines. Sampling followed a two-stage process: (1) 15 communities were selected to reflect urban communities, semiurban communities, and rural settings; and (2) the respondents were selected using a simple random sampling method. Because no structured form of housing exists (i.e., streets are typically unnamed, houses are not numbered, and household registration data are nonexistent), we designed the following sampling method.

In each village, we chose a starting point that was generally a central location in the village, such as a school, a community center, a district clinic, or a police station. We then selected the house closest to the starting point, and then chose every other house after until we obtained a sample size of approximately 25 respondents for each study area.

We surveyed 400 households by using the head of each household as the respondent.

### Data collection

We used a cross-sectional and pretested structured interviewer questionnaire for data collection. A pilot study was conducted on 50 respondents from different villages in St. Vincent and the Grenadines. We adjusted some of the survey questions based on the results obtained from the pilot study. The interviewers attended a 2-day training workshop before the pilot study, and participated in the pilot study to familiarize themselves with the instrument and bidding game technique. This also familiarized them with the concept of NHI, so that they could answer any questions from the respondents and explain the services to them.

To collect relevant information, the heads of each household from the randomly selected households in the 15 communities were interviewed. The questionnaire included sections on demographics, education, income, and health care use. As the participants were interviewed, the interviewers completed the questionnaires.

The interviewers described the purpose of the study, and asked the respondents whether they consented to participate. If the respondents consented, they were asked to sign an informed consent form. Informed consent has been provided by all participant before the interview. Of the 400 households we approached, all were willing to participate in the interview; thus, we achieved a response rate of 100%. The study was reviewed and approved by the National Ethics and Research Committee (NERC) of the MOHWE of St. Vincent and the Grenadines.

### Eliciting willingness to participate and pay

We presented a simple hypothetical NHI plan as shown below to the respondents and asked them should the government introduce a plan similar to this will they be willing to accept and participate in the plan:

“Hospitalization expenses incurred for medical or surgical treatment for illness/disease and injury up to ECD$ 5000 per year and person and reimbursement of cost for general practitioners and specialist, prescribed drugs and laboratory services.”

We used a contingent valuation approach to determine the willingness to pay. For this survey, we used a double-bounded dichotomous choice elicitation method. The respondents were presented with a hypothetical situation describing a potential NHI product. We proposed a basic package that covered hospitalization and expenses incurred for medical or surgical treatment and injury up to EC$5,000 (US$1,852) per year for every person, as well as a full reimbursement of the cost for the physician (general practitioner or specialist) office visit and all prescribed drugs and laboratory services. This value was obtained after consultation with personnel in the private health insurance sector in St. Vincent and the Grenadines. The maximum value was based on the per-disability maximum amount under the basic coverage package offered by one of the largest insurance companies in the country that included hospitalization, prescription drugs, surgical treatment, physician office visits, as well as specialist consultation and maternity benefits. The proposed NHI plan that was presented to the respondents was similar. We used this method because data are nonexistent regarding the annual cost of medical expenses per person in St. Vincent and the Grenadines.

**Figure 1:**
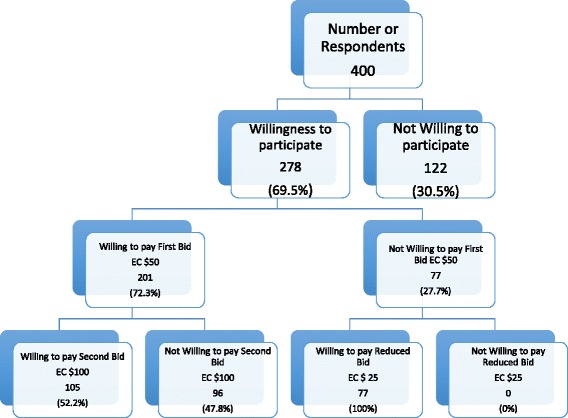
**Summary Statistics to double-bounded dichotomous choice questions.**

Using the dichotomous choice elicitation method, we asked each respondents if he or she was willing to participate in the proposed NHI if offered by the government and a positive premium is required. If the respondents answered “yes” then we considered them willing to participate in the NHI plan, before asking them whether they were willing to pay and how much they were willing to pay. We then presented the first bid of EC $50 and asked if they were willing to pay this as a monthly premium for themselves (Figure 1). If they answered, “yes” to the first bid, then we declared a second higher bid of EC $100 before asking them if they were willing to pay for that bid (“yes”/”no”). If the respondent answered “no” to the initial bid, then we proposed a second lower bid of EC$25. If they answered “no” to both the first and the second bids, then they were asked to mention the maximum amount that they were willing to pay. If they also answered, “yes” to the first and the second bids a maximum amount that they will be willingness to pay was also sought. If they answered, “yes” to the first bid and “no” to the second bid, a maximum amount that they were willingness to pay was also asked. In our sample, all the respondents who said that they were willing to participate (278 of 400) were all willing to pay for at least the second lower bid (EC$25).

### Data analysis

Chi-squared analysis was conducted to examine the influence of independent variables on the willingness to participate. We then performed multiple logistic regression to explore the factors associated with the willingness to pay for the proposed NHI. Significance was set at 5%. All analyses were performed using STATA MP 12.

## Results

Table 1 lists the hypothesized independent variables to describe the determinants of the willingness to participate and the willingness to pay for the proposed NHI plan in St. Vincent and the Grenadines.

**Table 1:** **Description of the independent variables hypothesized to explain willingness to participate and pay for National Health Insurance in St. Vincent and the Grenadines**

**Variables**	**Explanation**	**Measurement**	**Hypothesized relationship with willingness to pay**
Age	How old the respondents are in years	0 = Less than 30	Younger people will be less willing to pay than older ones
		1 = 31 - 45	
		2 = 46 – 60	
		3 = More than 60	
Sex	Whether the respondent is male or female	0 = male	Males will be more willing to pay than females
		1 = female	
Marital Status	Characteristic of respondent marital status	0 = Married	Respondents who are married have a greater willingness to pay
		1 = Single/Divorced/Widowed	
Total number of people in household:	The number of residents within each household	1 = 2 or less	Higher number of household residents will lead to higher willingness to pay
		2 = 3 – 5	
		3 = More than 5	
Geographic location	Whether the respondent resides in a rural or urban area	1 = rural area	Residents living in urban areas will be more willing to pay than those in rural areas
		2 = sub-urban area	
		3 = Urban area	
		4 = Grenadines	
Education level	Measures the highest level of education attained by the respondents	0 = Primary school completed	The greater the level of education attained the greater the willingness to pay will be
		1 = Secondary school completed	
		2 = Completed tertiary level education	
Individual Monthly Income	The gross monthly salary of the respondent in Eastern Caribbean Dollar (USD $1 = ECD $2.70)	0 = $500 or less	People who earn more will be more willing to pay
		1 = $501 - $1000	
		3 = More than $1 000	
Employment status	Whether the respondent is employed or not	0 = Not employed	People who are employed will be more willing to pay
		1 = Employed	
		2 = Self-employed	
		3 = Retired	
State of health	Respondents self rated health status	4 = Excellent	Respondents who are in better health will be less willing to pay than those in worse health
		3 = Very Good	
		2 = Good	
		1 = Fair	
		0 = Poor	
Health insurance ownership	If the respondents has health insurance or not	0 = No	Respondents with health insurance will be more willing to pay than those without
		1 = yes	
Level of satisfaction with the public health care system	Measures the respondent level of satisfaction with the public health care system	0 = Not satisfied	Respondents who are satisfied will be less likely to pay than those who are dissatisfied
		1 = Neutral	
		2 = satisfied	

Of the 400 randomly selected respondents, 278 (69.5%) agreed to participate in the offered NHI plan if a positive premium must be paid. From all of the surveyed communities, more than 50% of the respondents were willing to participate in the proposed NHI plan (Table 2). Kingstown, the capital, reported the highest willingness to pay (92.3%), whereas the rural village of Barrouallie reported the lowest willingness to pay (56.3%). Overall, the urban areas had a higher rate of willingness to participate compared with the rural villages. The mean percentage of respondents who were willing to participate according to geographic location was 82.1% for the rural communities, 72.3% for the suburban villages, 66.2% for rural communities, and 66.7% for the Grenadine Islands.

**Table 2:** **Percentage of participants from the study areas who were willing to participate in the proposed NHI**

**Location**	**Villages**	**Willing to participate (%)**	**Mean % willing to participate**
URBAN	Cane Garden	71.9%	
	Kingstown	92.3%	82.1%
SUB-URBAN	Belair/Fountain	74.2%	
	Campden Park	80.0%	72.73%
	Calliaqua	64.0%	
RURAL	Barrouallie	56.3%	66.18%
	Calder	85.0%	
	Clare Valley	60.0%	
	Enhams	68.0%	
	Georgetown	66.7%	
	Layou	74.1%	
	Richland Park	59.3%	
	Sandy Bay	60.0%	
GRENADINES	Bequia	62.5%	66.65%
	Union Island	70.8%	

Figure 1 shows the summary statistics of the responses to the double-bounded dichotomous choice questions. Of the respondents who were willing to participate, 72.3% (n = 201) were willing to accept the first bid of EC$50 monthly per person, whereas 22.7% (n = 77) were unwilling to pay this first bid. When the bid increased to EC$100 monthly per person, 52.2% (n = 105) of those willing to pay the first bid were also willing to pay this second bid, whereas 47.8% (n = 96) were unwilling to pay. Of the respondents who were not willing to pay the first initial bid of EC$50, when the bid was reduced to EC$25 monthly per person, all 77 (100%) indicated that they were willing to pay for this lowered bid. The average willingness to pay was EC$77.83 monthly per person for all of the respondents who were willing to participate in the NHI plan.

Table 3 shows the factors associated with the willingness to participate under the assumption of a positive premium. The chi-squared analysis showed that age, education, income, employment, health status, health insurance ownership, and the level of satisfaction with the existing public health care system were all statistically significant at 0.01%. People were less willing to participate with increasing age. Of those who were younger than 30 years, 84.52% were willing to participate. Respondents who were 65 years and older were less willing to participate (22.80%). Although the gender of the respondents was not statistically significant, the male respondents were more willing to participate than their female counterparts. Marital status, the number of household members, and location were also not statistically significant in the analysis.

**Table 3:** **Frequency distribution and chi-square analysis of the willingness to participate in the proposed NHI**

**Variables**	**Willingness to participate (n = 278)**	**Not willing to participate (n = 122)**	**Total (n = 400)**	**Chi** ^**2**^	**P value**
**Demographic Characteristics**					
**Age**					
<30 years	71 (84.52%)	13 (15.48%)	84 (21.00%)		
31- 45 years	106 (77.94%)	30 (22.06%)	136 (34.00%)	35.77	<0.001 ***
46 – 60 years	57 (65.52%)	30 (34.48%)	87 (21.75%)		
>60 years	44 (22.80%)	49 (77.2%)	93 (23.25%)		
**Sex**					
Male	145 (72.86%)	54 (27.14%)	199 (49.75%)	2.11	0.146
Female	133 (66.17%)	68 (33.83%)	201 (50.25%)		
**Marital status**					
Married	114 (65.14%)	61 (34.86%)	175 (43.75%)	2.79	0.095
Single/Divorced/widowed	164 (72.89%)	61 (27.11%)	225 (56.25%)		
**Number of household members**					
2 or less	99 (65.56%)	52 (34.44%)	151 (37.75%)	3.97	0.137
3 - 5	137 (74.46%)	47 (25.54%)	184 (46.00%)		
More than 5	42 (64.62%)	23 (35.38%)	65 (16.25%)		
**Location**					
Rural	139 (62.26%)	74 (34.74%)	213 (53.25%)		
Sub-urban	59 (72.84%)	22 (27.16%)	81 (20.25%)	7.23	0.065
Urban	48 (82.76%)	10 (17.24%)	58 (14.50%)		
Grenadines	32 (66.67%)	16 (33.33%)	48 (12.00%)		
**Socio-economic status**					
**Education**					
Primary	93 (54.71%)	77 (45.29%)	170 (42.50%)		
Secondary	91 (71.65%)	36 (28.35%)	127 (31.75%)	40.84	<0.001 ***
Tertiary	94 (91.26%)	09 (8.74%)	103 (25.75%)		
**Income**					
< $500	46 (42.20%)	63 (57.80%)	109 (27.25%)		
$501 - $1000	81 (71.05%)	33 (28.95%)	114 (28.50%)	59.32	<0.001 ***
> $1000	151 (85.31%)	26 (14.69%)	177 (44.25%)		
**Employment**					
Employed	198 (84.62%)	36 (15.38%)	234 (58.50%)		
Self-employed	44 (58.67%)	31 (41.33%)	75 (18.75%)	68.38	<0.001 ***
Not employed	17 (43.59%)	22 (56.41%)	39 (9.75%)		
Retired	19 (36.54%)	33 (63.46%)	52 (13.00%)		
**Health**					
**Self-rated health status**					
Excellent	11 (44.00%)	14 (56.00%)	25 (6.25%)	29.37	<0.001 ***
Very good	60 (56.60%)	46 (43.39%)	106 (26.50%)		
Good	96 (70.07%)	41 (29.93%)	137 (34.25%)		
Fair	58 (82.85%)	12 (1.71%)	70 (17.50%)		
Poor	53 (85.48%)	09 (14.52%)	62 (15.50%)		
**Other**					
**Health insurance ownership**					
Yes	78 (97.5%)	02 (2.50%)	80 (20.0%)	36.99	<0.001 ***
No	200 (62.50%)	120 (37.50%)	320 (80.00%)		
**Level of satisfaction with the public health care system**					
Not Satisfied	90 (84.91%)	16 (15.09%)	106 (26.50%)	18.40	<0.001 ***
Neutral	31 (73.80%)	11 (26.19%)	42 (10.50%)		
Satisfied	157 (62.30%)	95 (37.70%)	252 (63.00%)		

*** p < 0.001.

A rising level of education and income accompanied a greater willingness to participate. Employed respondents were more willing to participate than those who were self-employed, unemployed, or retired. People in poor health were more willing to participate than those with excellent and good health, and respondents who had private health insurance were more willing to participate than those who did not. Regarding satisfaction with the public health care system, respondents who were unsatisfied were more willing to participate than those who said that they were satisfied.

We performed multiple logistic regression (Table 4) to explore the factors associated with the participants’ willingness to participate in an NHI program. The factors that were statistically significant in the chi-squared analysis were used in the model. Education, income, and employment were highly collinear; thus, we retained only income in the model.

**Table 4:** **Willingness to pay for the proposed NHI (n = 400) – Logistic regression showing the relationship between the independent variable and willingness to pay**

**Parameter**	**Odds ratio**	**p-value**
Intercept	3.24	0.062
Age (reference < 30 years)		
31- 45 years	0.60	0.205
46 – 60 years	0.41	0.032
>60 years	0.26	0.002
Sex (reference: male)	1.21	0.460
Income (reference: < EC $500)		
$501 - $1000	2.93	0.001
> $1000	3.87	<0.001
Health Status (reference: poor)		
Excellent	0.74	0.647
Very good	0.64	0.358
Good	0.62	0.301
Fair	0.89	0.829
Insurance (reference: no insurance)	12.93	0.001
Satisfaction (reference: not satisfied with the public health care system)		
Neutral	0.41	0.075
Satisfied	0.58	0.123

Age, income, and people who already had health insurance were significant factors associated with the willingness to participate. The results indicate that a higher income increases the willingness of respondents to participate in an NHI program. After we controlled for all other variables, respondents who were older (>60 years old and 45–60 years old) were less likely to participate than respondents aged 30 years and younger. Respondents with a higher income were more likely to participate than respondents with a lower income. Respondents who earned between EC$501 and $1000 and more than $1000 were more likely to be willing to participate than those who earned $500 and less. Respondents who had a health insurance plan of some form were more likely to be willing to participate than those who had no health insurance.

## Discussion

For a health insurance program to be established, health sectors must undergo reforms at the national level. Health sector reform is not easy, and even developed countries face delays and challenges when attempting to reform their respective health sectors and establish a social health insurance system [28-30]. Developing countries have even greater challenges when seeking to adopt a health insurance program. The findings of this study can help enhance people’s understanding of the willingness to participate and pay, in addition to factors that must be considered when planning and implementing such a system.

The finding that 69.5% of the respondents are willing to participate in the proposed plan has significant implications if a NHI program is to be established. This result may indicate to policy makers that people generally accept such a system. This study can inform policy makers to devise a plan that promotes maximum participation by the people. Identifying the factors that are positively associated with the willingness to participate can facilitate establishing and implementing a NHI framework.

Because older people are at a greater risk of adverse health events, we hypothesized that older respondents are more likely to be willing to participate compared with younger respondents. However, we found that the respondents in the age group >60 years and those aged 45 to 60 years were less likely to be willing to participate than those who were 30 years old and younger. Barnighausen et al. [31] found that the willingness to pay decreases with age. He suggested that one possible explanation might be that older respondents believe that they can depend on their children should they become ill.

Income was a significant variable in our study. Respondents with more income were more likely to be willing to participate than those who earned less. Most studies in developing countries found income to be a significant factor in determining willingness to pay for health insurance [32,33]. Our findings suggest that people with a higher income are more likely to be willing to participate than those from a lower income bracket. This may suggest people who are employed in the formal sector where income may be a bit higher may be the ones with greater interest in a NHI plan. This is import to consider this since certainty of income from public sector or more formal employment may make it an easier source of payment of NHI. As a public insurance system what can happen is that there can be income redistribution where people of higher SES can contribute more especially in a developing country where unemployment is high and the informal sector employs a great part of the labor force.

It may be reasonable to establish such a plan in phases. In the first phase of the plan, enrolling people who are employed in the public sector (i.e. government workers on a fixed income) may be a reasonable option as to ensure maximum participation in NHI.

The findings that having health private insurance is a significant factor associated with the willingness to participate may be due to people with health insurance already exposed to the insurance system, thus understanding its importance. When the respondents were asked about having health insurance, only 20% of them reported that they had some form of health insurance. A 2009 report stated that only 9.4% of the St. Vincent and the Grenadines population reported having health insurance [11]. This difference between the 2009 report and our study may be attributed to some of the respondents belong to credit unions and other Cooperates that offer financial assistance in the event of sickness when a member cannot fulfill medical care cost. Therefore, they may have reported this as having health insurance. The difference between the years of study may also be a reason.

Although not statistically significant, respondents who were satisfied with the system were less likely to be willing to participate than those who were not. Respondents who were unsatisfied may believe that health insurance may be a way to improve health care; therefore, they were more likely to be willing to pay. Respondents who expressed satisfaction may have believed that they were receiving what they wanted from the system, and this, may not have felt obligated or inclined to pay health insurance. Health insurance may thus be a way to improve the quality of care provided by public health care facilities.

The average amount that the respondents were willing to pay can contribute positively to financing the health sector. The per-capita health expenditure in St. Vincent and the Grenadines was US$ 310 in 2011. If the NHI program is to be implemented, using our willingness to participate as an indicator approximately 71, 868 people will be expected to participate. Based on the labor force data, if approximately 67.5% are willing to participate and to pay then approximately 30, 000 workers would enroll. This can then generate close to 1 million USD annually in premium collected provided that everyone who enrolls pay the average maximum willingness to pay amount as found in this study.

Our study showed that SES does play a significant role in the willingness to participate and pay for health insurance. This means that only those with a greater economic capacity would acquire private insurance or currently have private health insurance. This may be reflected in the low percentage of the population that is enrolled in private health insurance. This could lead to the splitting the supply of medical services among different sub-population groups with different economic capacity [34]. Private health insurance may seem to be an option because of the insufficient capacity in implementing a public system or because not many studies have been done in exploring a NHI in the Caribbean region. This study shows people’s interests in NHI and their willingness to participate. With high out of pocket spending and SES being a significant determining factor the pooling of resources via NHI may be a viable alternative for financing health care.

If the government is to maintain its budgetary allocation to the sector coupled with NHI contributions, then the financing of the health sector can be improved, thus leading to enhanced services and providing a pathway for universal health care.

There were some limitations in this study. As with most contingent valuation studies, the elicitation technique is always subjected to bias and the assignment of the first bid is also a significant factor. We noted that after our first bid was lowered, all the respondents’ accepted the lowered bid. It could be that our starting bid was too low in the first place or that we did lower the bid too low. Another limitation is that no increase benefit from the NHI package came with an increase in the bid amount and so respondent could have accepted the minimum bid knowing that they would get the same benefits.

Interviewer bias is also to be considered as they can convince respondents to participate and to choose the bids that they though would be more appropriate or the respondents. Respondents may have equated participation with paying and thought that once they have answered yes that they were willing to participate that they were obligated to choose a positive premium amount to pay. Also, we only collected data on individual willingness to participate and pay. Factors like family composition do influence willingness to pay but we did not collect data on the willingness to pay for the entire household based on family composition and size.

This is the first report on the willingness to participate and pay study on NHI in St. Vincent and the Grenadines. It is also the first study at the micro economic level to assess participation in an NHI program in St. Vincent and the Grenadines. All feasibility studies conducted by the government of St. Vincent and the Grenadines used macro economic data [35-37]. Because of similarities in the background of the Caribbean countries, our results can be generalized as a reference for other Caribbean countries wishing to implement NHI.

## Conclusion

If developing countries like St. Vincent and the Grenadines intend to adopt universal health coverage, then alternative ways to finance the health sector must be sought. An implemented NHI program can serve as a reliable income for health care providers, help in cost recovery for the health sector and protect the poor against high out of pocket spending.

Government must raise awareness among low-income earners and those without existing health insurance to increase the demand for NHI coverage. Therefore, people with lower socioeconomic status must be engaged from the start of and throughout the development process to enhance their understanding of and participation in the plan.

## References

[1] National Census Report. Government of St. Vincent and the Grenadines; Statistical Office. Ministry of Finance and Economic Planning. 2001.

[2] Inter-American Development Bank, Compete Caribbean, Private Sector Assessment of St. Vincent and the Grenadines. 2013

[3] US Department of State. 2013 Investment Climate State. St. Vincent and the Grenadines: http://www.state.gov/e/eb/rls/othr/ics/2013/204724.htm

[4] Huff-Rousselle M, Lalta S, Fiedler J (1998). Health financing policy formulation in the Eastern Caribbean. Int J Health Plann Manag.

[5] International Social Security Association (ISSA) (2006). Social Security in the Caribbean: Management, Health Care, Harmonization.

[6] Huff-Rousselle M, Roberts K, Richards C, Lalta S (1995). Preliminary Discussion on National Health Insurance in Grenada and St Vincent and the Grenadines.

[7] Lalta S (1996). National Health Insurance and Health Security in St Vincent and the Grenadines.

[8] Pan American Health Organization (PAHO). 2012. Health situation analysis and trend summary. http://www1.paho.org/english/dd/ais/cp_670.htm. Washington: Pan American Health Organization.

[9] (UNDP), UNDP, St. Vincent and the Grenadines Health System and Private Sector Assessment, 1996.

[10] World Health Organization (WHO). 2011. Global Health Observatory. http://apps.who.int/ghodata/. Geneva: World Health Organization.

[11] Caribbean Development Bank (CDB) (2009). St. Vincent and the Grenadines Country Poverty Assessment 2007/2008: Living Conditions in a Caribbean Small Island Developing State.

[12] World Health Organization (WHO). 2012. Health financing: Health expenditure ratios by country. WHO. Global health observatory data repository. http://apps.who.int/gho/data/node.main.485. Geneva: World Health Organization.

[13] Government of St. Vincent and the Grenadines (GOSVG) (2011). House of Assembly Estimates of Revenue and Expenditure for 2011.

[14] Government of St. Vincent and the Grenadines. Ministry of Health, Wellness and the Environment. Monitoring and Evaluation of the Health Sector Report. 2013.

[15] Kirigia J, Sambo L, Nganda B, Mwabu G, Chatora R, Mwase T (2005). Determinants of health insurance ownership among South African women. BMC Health Serv Res.

[16] Lang H, Lai M (2008). Willingness to pay to sustain and expand national Health Insurance services in Taiwan. BMC Health Serv Res.

[17] Wang H, Yip W, Zhang L, Wang L, Hsiao W (2005). Community-based health insurance in poor rural China: the distribution of net benefits. Health Policy Plan.

[18] Asenso-Oykere W, Osei-Akoto I, Anum A, Appiah E (1997). Willingness to pay for health in a developing economy. A pilot study of the informal sector of Ghana using contingent valuation. Health Policy.

[19] Dong H, Kouyate B, Cairns J, Mugisha F, Sauerborn R (2003). Willingness-to-pay for community-based insurance in Burkina Faso. Health Econ.

[20] Dong H, Kouyate B, Cairns J, Sauerborn R (2004). Differential willingness of household heads to pay community-based health insurance premia for themselves and other household members. Health Policy Plan.

[21] Dong H, Kouyate B, Snow R, Mugisha F, Sauerborn R (2003). Gender’s effect on willingness-to-pay for community-based insurance in Burkina Faso. Health Policy.

[22] Dong H, Mugisha F, Gbangou A, Kouyate B, Sauerborn R (2004). The feasibility of community-based insurance in Burkina Faso. Health Policy.

[23] Asgary A, Willis K, Taghvaei A, Rafeian M (2004). Estimating rural households’ willingness to pay for health insurance. European J Health Econ.

[24] Donfouet P, Makaudze E, Mahieu P, Malin E (2011). The determinants of the willingness-to-pay for community-based prepayment scheme in rural Cameroon. Int J Health Care Finance Econ.

[25] Dong H, Kouyate B, Cairns J, Sauerborn R (2003). A comparison of the reliability of the take-it-or-leave-it and the bidding game approaches to estimating willingness- to-pay in a rural population in West Africa. Soc Sci Med.

[26] Onwujekwe O, Okereke E, Onoka C, Uzochukwu B, Kirigia J, Petu A (2010). Willingness to pay for community-based health insurance in Nigeria: do economic status and place of residence matter?. Health Policy Plan.

[27] Shackley P, Donaldson C (2002). Should we use willingness to pay to elicit community preferences for health care? New evidence from using a ‘marginal’ approach. J Health Econ.

[28] Bogg L, Hengjin D, Keli W, Wenwei C, Diwan V (1996). The cost of coverage: rural health insurance in China. Health Policy Plan.

[29] Cooper MH (1994). Jumping on the spot to health reform New Zealand style. Health Econ.

[30] Flynn ML, Chung YS (1990). Health care financing in Korea: private market dilemmas for a developing nation. J Public Health Policy.

[31] Barnighausen T, Liu Y, Zhang X, Sauerborn R (2007). Willingness to pay for social health insurance among informal sector workers in Wuhan, China: a contingent valuation study. BMC Health Serv Res.

[32] Dror DM, Radermacher R, Koren R (2007). Willingness to pay for health insurance among rural and poor persons: field evidence from seven micro health insurance units in India. Health Policy.

[33] Bustamante A, Ojeda G, Castaneda X (2008). Willingness to pay for cross-border Health Insurance between the United States and Mexico. Health Aff.

[34] Frenk J, González-Pier E, Gómez-Dantés O, Lezana MA, Knaul FM (2006). Comprehensive reform to improve health system performance in Mexicoi. Lancet.

[35] St. Vincent and the Grenadines. National Health Insurance Project. Final Report. The Cost of Health Services in St. Vincent and the Grenadines. 1997.

[36] St. Vincent and the Grenadines. National Health Insurance Project. Final Report. Macroeconomic and Socioeconomic Profiles. 1999a.

[37] St. Vincent and the Grenadines. National Health Insurance Project. Final Report. Policy Framework of the NHIP. 1999b.

