# Erratum to: Willingness to participate and Pay for a proposed national health insurance in St. Vincent and the grenadines: a cross-sectional contingent valuation approach

**DOI:** 10.1186/s12913-016-1322-9

**Published:** 2016-02-23

**Authors:** Rosmond Adams, Yiing-Jenq Chou, Christy Pu

**Affiliations:** 1 Ministry of Health, Wellness and Environment, Ministerial Building, Halifax Street, Kingstown, St. Vincent and the Grenadines; 2 Department of Public Health, National Yang Ming University, 155, Sec.2, Linong Street, Taipei, 112 Taiwan

Upon publication of this article [1] it has been noted that the acknowledgement section was missed out. It should read as below:
